# Using a double-channel gastroscope reduces procedural time in performing gastric endoscopic submucosal dissection

**DOI:** 10.12669/pjms.323.9743

**Published:** 2016

**Authors:** Xu Li Hua, Li Liang Jun, Zhou Chuan Wen, Ji Ying Lin, Tian Ye, Li Xue Liang

**Affiliations:** 1 Xu Li Hua, Department of Gastroenterology, The First Affiliated Hospital of Nanjing Medical University, Nanjing (210029), China; 2 Li Liang Jun, Department of Gastroenterology, Huai’an First People’s Hospital, Nanjing Medical University, Huai’an (223300), China; 3 Zhou Chuan Wen, Department of Gastroenterology, Huai’an First People’s Hospital, Nanjing Medical University, Huai’an (223300), China; 4 Ji Ying Lin, Department of Gastroenterology, The First Affiliated Hospital of Nanjing Medical University, Nanjing (210029), China; 5 Tian Ye, Department of Gastroenterology, The First Affiliated Hospital of Nanjing Medical University, Nanjing (210029), China; 6 Li Xue Liang, Department of Gastroenterology, The First Affiliated Hospital of Nanjing Medical University, Nanjing (210029), China

**Keywords:** Double-channel gastroscope, Endoscopic submucosal dissection, Endoscopic ultrasound, Gastric antrum lesion

## Abstract

**Objective:**

Complications are important determining factors for safety of endoscopic submucosal dissection (ESD). ESD of large lesions is associated with increased procedural time. This study investigated whether double-channel gastroscope could be used to reduce procedural time in gastric antrum ESD.

**Methods:**

A retrospective cohort study of 46 patients with one gastric antrum lesion resected by ESD was conducted between January 2013 and December 2015. The diameter of a lesion was from 2cm to 4cm in 46 patients. EUS before ESD was used to evaluate the submucosal vascular structure and the location of lesion in gastric wall. Forty six lesions had ESD with either the ordinary gastroscope (OS group) (n=24) or the double-channel gastroscope (DC group) (n=22).

**Results:**

The mean procedural time was significantly lower in the DC group than in the OS group (49.1 minutes vs. 20.5 minutes, p=0.04). There were no significant differences in submucosal injection frequency, specimen size, *en bloc* resection rate and perforation rate between the two endoscopic groups. There was no recurrence in any case during the follow-up period.

**Conclusions:**

Our data suggest that ESD utilizing double-channel gastroscope may provide a better platform for quicker ESD with equal safety.

## INTRODUCTION

Endoscopic submucosal dissection (ESD) has been gaining wide-spread acceptance as a treatment for gastric neoplasms, including early gastric cancer, GIST (gastrointestinal stromal tumor), and so on, because of its high rate of *en bloc* resection.^1^-^3^ The “grasp-and-snare” technique in EMR, using snare and injection needle devices, has been proven to be safe and effective.^4^,^5^ Nonetheless, “grasp-and-cut” ESD can become a technically demanding and particularly time-consuming procedure in cases with large(2-4 cm) or giant (>4 cm) lesions, and there are still several unsolved problems associated with ESD using, such as high incidence of perforation, bleeding, and other complications.^3^

Adequate tissue tension and clear visibility of the tissue to be dissected by traction are important for effective and safe ESD. The double-channel gastroscope (DCG) has the advantages of simple structure, convenient operation, multiple functions, economic and practical.^6^,^7^ The “grasp-and-cut” technique can be used to perform ESD with good outcomes and low complication rates.

The primary aim of our retrospective study was to evaluate whether the use of a DCG for ESD in the stomach is related to a significant reduction in the procedural time compared to the use of an ordinary gastroscope (OS). The secondary aims were to record and compare other ESD parameters such as injection frequency, specimen size, adverse effects, perforation of the procedure dependent on DCG or OS use.

## METHODS

This study was commenced after obtaining approval from the Ethics Committee at The First Affiliated Hospital of Nanjing Medical University and Huai’an First People’s Hospital in January 2013. The destination for ESD with curative intent was clinically diagnosed adenoma, intramucosal cancer or GIST and fulfillment of the criteria of the Japanese Gastric Cancer Treatment Guidelines.^8^ ***inclusion criteria***^9^ were: 1) age between 20-80 years old; 2) the lesion located in the gastric antrum; 3) having received EUS prior to ESD; and 4) having provided written informed consent. The ***exclusion criteria***^10^ for patients were: 1) resection of multiple lesions during a single procedure, 2) evidence of a scar, which made it difficult to identify the submucosal vascular structures by EUS, 3) serious liver/kidney dysfunction or hematological disorder, 4) receiving antiplatelet or anticoagulation therapy the past week, or 5) inappropriate for participation in this study.

This was a retrospective cohort study. Between January 2013 and December 2015, ESD was performed through a DCG or an OS for 485 gastric lesions in 471 consecutive patients at The First Affiliated Hospital of Nanjing Medical University and Huai’an First People’s Hospital, China. Forty six patients were enrolled in the study, and a thorough explanation of the procedure was provided to the patients. There were three cases of early gastric cancer, 18 cases of moderate dysplasia, 14 cases of severe dysplasia, 11 cases of GIST. Ages of the 46 patients ranged from 21 to 78 years old, and the mean age was 46.5. The history of presenting problems was upper abdominal pain in 13 patients (28.1%), abdominal distension in 6 (13.0%), and black stools in 6(13.0%), while the rest of the subjects were discriminated incidentally with endoscopy without specific clinical symptoms.

EUS was performed with a UM-2000 system (Olympus Optical, Tokyo, Japan). A deaerated water-filling method for the observation and a 20MHz miniature probe (Olympus Optical, Tokyo, Japan) was used.

ESD was operated by skilled endoscopists who had performed more than 100 patients of gastric ESD. GIFQ260J or GIF2TQ260M endoscope (Olympus Optical, Tokyo, Japan) was used during the procedures, plus a hook knife (Olympus Optical, Tokyo, Japan), insulated-tip knife (KD-611L, IT2; Olympus, Japan), an ICC200 or VIO300D high-frequency apparatus (ERBE, Tübingen, Germany), and CR4500 CO_2_ Regulation Unit (AGS MedTech, Hangzhou, China). A short, transparent cap (ND-201-11802, Olympus, Japan) was attached to the tip of the gastroscope to furnish a constant endoscopic view and to apply tension to the connective tissue for dissection. All the endoscopic procedures were executed under general anesthesia. The solution for the submucosal injection was prepared by adding small amounts of epinephrine and indigo carmine to 0.9% physiological saline (5ml indigo carmine, 1ml epinephrine and 100ml physiological saline). After marker dots were placed around the lesion, a solution (above-mentioned) was injected around the lesion to lift it off the muscularis propria layer, and the incision was started outside the marker dots using the hook knife /the insulated-tip knife. Followed by the submucosa identified under direct vision, *en bloc* resection of the lesion was separated from the submucosa. If lesion involved the muscularis propria layer, it was peeled with the hook knife /the insulated-tip knife to the muscularis propria layer along the edge of the lesion.^9^ Intraoperative bleeding was controlled by coagulation hemostasis with the tip of the knife (swift coagulation, effect 4, 40W) for mild bleeding or with hemostatic forceps (Olympus Optical, Japan; soft coagulation, effect 4, 50W) for moderate bleeding. When hemostasis was difficult using these procedures, clips (Olympus Optical, Japan) were used to hold the vessel for hemostasis. A damaged muscle layer was clipped for plication at the discretion of the operator.

This study investigated the following: (1) the procedure time (defined as the time from endoscope insertion to removal), (2) injection frequency, (3) incidence of perforation which was defined as the detection of free air on postoperative X-ray, (4) incidence of postoperative fever of ≥38°C, (5) incidence of postoperative bleeding.

### Pathologic Evaluation

Pathological examination of the resected specimen was performed using parallel 2mm thick sections stained with hematoxylin and eosin. Lateral and vertical margins negative for the lesion, and intramucosal cancer (m) or minute submucosal penetration (sm1, up to 500μm into the submucosal layer) with no venous or lymphatic invasion by microscopic tissue examination.^11^

### Follow-Up

Endoscopy and EUS were followed up for the patients at 1, 2, 6, and 12 months after the last endoscopic resection and yearly thenceforth.

### Statistical Analysis

Data were analyzed using the SPSS version 16.0 (SPSS Inc., Chicago IL, USA). Continuous data were compared using the unpaired Student *t*-test or Mann-Whitney U-test, as appropriate. Categorical variables were examined using the corrected chi-square or two-sided Fisher exact test. P-values of less than 0.05 were considered statistically significant.

## RESULTS

En bloc resection was performed by ESD, EUS revealed the location of a lesion in gastric layer in every case. No significant intergroup difference was observed in the demographic characteristic. (Table-I). All lesions were successfully resected by a single experienced endoscopist through double-channel gastroscope or ordinary gastroscope. And all lesions were affirmed histopathologically. All parameters (procedure time, specimen size, injection frequency, *en bloc* resection status and gastric perforations) were successfully recorded by an independent observer for each procedure.

**Table-I:** Comparison of clinicopathological factors between the two groups.

*Group*	*OS group*	*DCG group*	*P value*
Patients	24	22	
Gender (male : female)	13:11	12:10	0. 23
Mean age (years±SD^∆^)	47.0±8.8	45.2±10.4	0.14
Location of the lesion (A :P : L:S)	3:7:13:1	2:5:14:1	0.92
Median maximum diameter of tumor (mm±SD^∆^)	24.4±7.6	25.7± 8.7	0.75

Depth of invasion (M/SM/MP) 6/14/4 7/10/5 0.70

M:mucosal, SM: submucosal, MP: muscularis propria

OS group, ordinary gastroscope group;

DC group, double-channel gastroscope group;

Δ SD: standard deviation.

A (region), the anterior wall of the gastric antrum;

P (region), the posterior wall of the antrum;

L (region), the greater curvature side of the antrum;

S (region), the small curved side of the antrum.

The mean procedural time was significantly lower in the DCG group than in the OS group (20.5±19.4minutes vs. 49.1±18.5minutes, p=0.04) (Table-II). There were no significant difference between the two groups of the injection frequency, *en bloc* resection rate, perforation rate, incidence of postoperative bleeding or fever, and the median hospital stay.

**Table-II:** Comparison of outcome measures between the two groups.

*Group (n)*	*OS group (n=24)*	*DC group (n=22)*	*P value*
Procedure time (min±SD^∆^)	49.1±18.5	20.5±19.4	0.04
Injection frequency	4.5±0.6	2.5±0.6	0.88
En bloc resection %	100% (24/24)	100% (22/22)	-
Perforation %	4.1% (1/24)	4.5% (1/22)	0.55
Specimen size (mm±SD^∆^)	29.3±6.5	30.6±8.1	0.83
Incidence of postoperative bleeding (%)	2 (8.3)	1(4.5)	0.92
Incidence of postoperative fever (%)	1 (4.1)	1(4.5)	0.55
The median hospital stay	5.5±2.0	4.5±2.5	0.80

∆ SD: standard deviation. OS group, ordinary gastroscope group.

DC group, double-channel gastroscope group.

In all cases, 44(95.7%) wounds had healed at one month follow-up, the remaining 2 (4.3%) were completely recovered at two month follow-up. During the median 18-month (range: 10–40 months) follow-up period, there were no recurrences in any cases. The patients reported good quality of life, and no reflux, weight loss, poor appetite, intestinal adhesions, or other complications that usually occur after conventional open surgery.

## DISCUSSION

ESD is a well-established endoscopic technique for the treatment of larger (>2 cm) mucosal and submucosal neoplasms throughout the GI tract. Its use has been mainly in the stomach, and has also been in the esophagus, colon and rectum. Improving the visualization of the submucosal layer is important to get the more effectiveness and safety of ESD.^1^ Indigent visualization of the submucosal layer may need a more technically dissection, may result in a piecemeal resection, prolonged procedure, increased complications and so on.^1^-^3^ Usually there are two methods to get an adequate visualization of the submucosal layer. One is utilization of a submucosal injection solution and the other is tissue traction. The submucosal injection solution may has the potential of increasing the *en bloc* resection rate, decreasing the procedure time, or injection volume. However, there are still several limitations encountered, such as problem of tissue damage, smoke production, high price, and so on.^12^

Voudoukis et al. reported use of a double-channel gastroscope reduces procedural time in large left-sided colonic endoscopic mucosal resections.^6^,^7^ This double-channel gastroscope (DCG) has the potential to be applied and have the most impact in the ESD field of therapeutic endoscopy.^6^,^13^ Nishizawa et al.^14^ called it the “two-sword fencing” technique in ESD, which also saves the time of changing endoscopic devices. Simple and flexible methods with traction can make ESD easier and safer. The DCG use may improve the lifting of lesions and allow an improved dissection. DCG gives the option of placing both injection needle and insulated-tip knife in each of its working channels, using them tandemly according to endoscopist’s willingness for injection/grasp or cut. This technical advantage makes this method convenient to use and reduces the ESD time, thus facilitating the endoscopist in the study. Moreover, compared with OS use in ESD, DCG use provides similar safety and efficacy with regard to parameters such as *en bloc* resection rate, complications, specimen size, full-thickness gastric perforation and recurrence rates.

The bottom of the dissected mucosal layer is pushed and lifted up using the injection sheath through one channel to reveal the submucosal layer and ensure adequate traction, and submucosal dissection was conducted by an IT-knife through the other channel. On the contrary, it is sometimes difficult to control the traction direction. Hijikata et al.^15^ reported on ESD using the outer sheath of an injection needle, a double-channel scope is thicker, heavier, and more difficult to manipulate than a single-channel endoscope. Moreover, since the grasping forceps or the outer sheath is inserted through the endoscope, it moves synchronously with the endoscope, which sometimes makes it difficult to control the traction direction and to cut the submucosal layer of larger lesions.

There are still some limitations in our study. Firstly, it was a retrospective study. Secondly, our patient sample size was only moderate-sized (20-40mm) lesions. Thirdly, this was a double center study only.

In conclusion, use of a DCG for ESD can be a very convenient technique for endoscopists as it could reduce the procedural time in Performing Endoscopic Submucosal Dissection. Prospective randomized controlled studies are needed to verify our results.
